# A 60% Edible Ethanolic Extract of *Ulmus davidiana* Inhibits Vascular Endothelial Growth Factor-Induced Angiogenesis

**DOI:** 10.3390/molecules26040781

**Published:** 2021-02-03

**Authors:** Jeongho Park, Hyun-Ouk Kim, Kwang-Hyun Park, Myung-Bok Wie, Sun-Eun Choi, Jang-Hyuk Yun

**Affiliations:** 1 College of Veterinary Medicine and Institute of Veterinary Science, Kangwon National University, Chuncheon 24341, Korea; jhp@kangwon.ac.kr (J.P.); mbwie@kangwon.ac.kr (M.-B.W.); 2 Department of Biotechnology and Bioengineering, Kangwon National University, Chuncheon 24341, Korea; kimhoman@kangwon.ac.kr; 3 Biohealth-Machinery Convergence Engineering, Kangwon National University, Chuncheon 24341, Korea; 4 Department of Emergency Medical Rescue, Nambu University, Gwangju 62271, Korea; khpark@nambu.ac.kr; 5 Department of Emergency Medicine, Graduate School of Chonnam National University, Gwangju 61469, Korea; 6 Department of Forest Biomaterials Engineering, Kangwon National University, Chuncheon 24341, Korea

**Keywords:** angiogenesis, endothelial cell, proliferation, tube formation, migration, *Ulmus davidiana*

## Abstract

As abnormal angiogenesis is associated with exacerbation of various diseases, precise control over angiogenesis is imperative. Vascular endothelial growth factor (VEGF), the most well-known angiogenic factor, binds to VEGF receptor (VEGFR), activates various signaling pathways, and mediates angiogenesis. Therefore, blocking the VEGF-induced angiogenic response-related signaling pathways may alleviate various disease symptoms through inhibition of angiogenesis. *Ulmus davidiana* is a safe natural product that has been traditionally consumed, but its effects on endothelial cells (ECs) and the underlying mechanism of action are unclear. In the present study, we focused on the effect of a 60% edible ethanolic extract of *U. davidiana* (U60E) on angiogenesis. U60E inhibited the VEGF-mediated proliferation, tube formation, and migration ability of ECs. Mechanistically, U60E inhibited endothelial nitric oxide synthase activation and nitric oxide production by blocking the protein kinase B signaling pathway activated by VEGF and consequently inhibiting proliferation, tube formation, and migration of ECs. These results suggest that U60E could be a potential and safe therapeutic agent capable of suppressing proangiogenic diseases by inhibiting VEGF-induced angiogenesis.

## 1. Introduction

Angiogenesis is the process of forming new blood vessels from existing ones. As any dysregulation in angiogenesis is associated with various diseases, this process requires elaborate control [1,2,3]. In particular, uncontrolled angiogenesis around the tumor and in the retina may worsen cancer and retinopathy, respectively. As tumor cells need blood vessels to receive nutrients and oxygen, angiogenesis is common around tumor cells and facilitates cancer growth and metastasis [3]. Further, abnormal angiogenesis is common in retinopathy such as diabetic retinopathy (DR) and retinopathy of prematurity (ROP), which may lead to blindness owing to the dysfunction of the retina [4,5]. Therefore, preventing abnormal angiogenesis under disease conditions such as cancer or retinopathy may offer potential therapeutic benefits.

Vascular endothelial growth factor (VEGF) is the most well-known protein that increases endothelial cell (EC) proliferation and mediates angiogenesis [6,7]. VEGF specifically acts on the ECs of blood vessels [8] and binds to and activates the VEGF receptor (VEGFR) on ECs [9]. This process activates several downstream signaling pathways such as phosphoinositide 3-kinase/protein kinase B (Akt) and mitogen-activated protein kinase/extracellular signal regulated kinase (Erk) in ECs, thereby increasing angiogenesis [9,10,11]. Interestingly, VEGF level is known to increase in the serum or tissues of patients with various types of cancers [12,13,14] as well as in the retina, sera, or vitreous of patients with DR and ROP [15,16,17,18,19]. Blockade of VEGF expression is thought to serve as an effective treatment strategy through prevention of abnormal angiogenesis in these patients [20,21,22]. In particular, anti-VEGF therapy using a VEGF neutralizing antibody is adopted in patients with cancer, DR, and ROP; however, this treatment is very expensive and associated with various side effects [20,21,22,23]. Therefore, it is necessary to develop a cheaper and safer agent that could prevent angiogenesis induced by VEGF.

*Ulmus davidiana* is a deciduous broad-leaf tree widely distributed in the Orient. It is considered as safe and has been traditionally consumed in the form of food or drugs. *U. davidiana* is known to exhibit pharmacological properties such as antioxidant, anti-inflammatory, and anticancer effects [24,25,26], and its stem and root have been used to treat diseases such as edema, mastitis, cancer, inflammation, and rheumatoid arthritis [27]. *U. davidiana* extracted with methanol was shown to inhibit chick chorioallantoic membrane angiogenesis [28]. How *U. davidiana* extract directly affects EC function, is however, unclear. Further, the effect of *U. davidiana* on angiogenesis induced by VEGF and the underlying mechanism warrant further studies.

In this study, we evaluated the effects of a 60% edible ethanolic extract of *U. davidiana* (U60E) on VEGF-induced angiogenesis and investigated the underlying mechanism of action. We demonstrate that U60E prevented the proliferation, tube formation, and migration abilities of ECs by blocking the VEGF-induced Akt/endothelial nitric oxide synthase (eNOS)/nitric oxide (NO) pathway.

## 2. Results

### 2.1. U60E Reduces NO Production by Suppressing eNOS Activity in Human Umbilical Vein Endothelial Cells (HUVECs)

**Figure 1:**
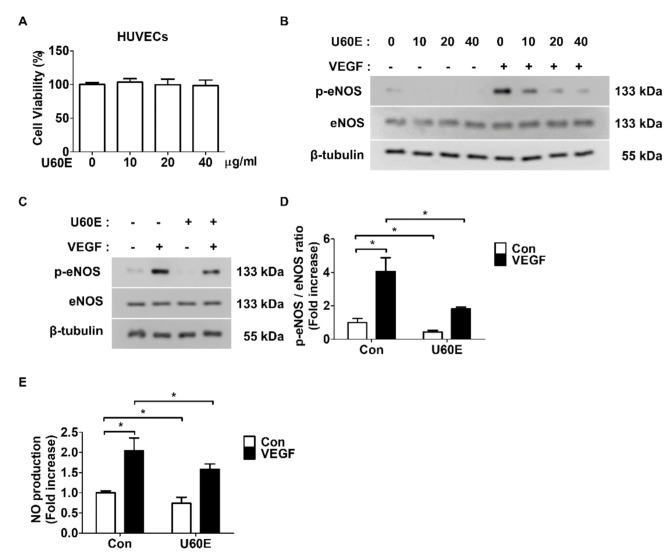
Effect of *U. davidiana* (U60E) on endothelial nitric oxide synthase (eNOS) activation and Nitric oxide (NO) production in Human Umbilical Vein Endothelial Cells (HUVECs). (**A**) HUVECs were treated with U60E for 24 h at indicated doses. The cell viability was determined by the MTT assay. The bar graph represents the means ± SD (*n* = 4). (**B**) HUVECs were treated with Vascular endothelial growth factor (VEGF) (20 ng/mL) and/or U60E (indicated doses) for 30 min. The phosphorylation of eNOS (p-eNOS) was determined by Western blot analysis. eNOS and β-tubulin were used as controls. (**C**) HUVECs were treated with VEGF (20 ng/mL) and/or U60E (20 μg/mL). The phosphorylation of eNOS (p-eNOS) was determined by Western blot analysis. (**D**) Quantitative densitometric analysis of Western blots in (**C**). The bar graph represents the means ± SD (*n* = 3). (**E**) HUVECs were treated with VEGF (20 ng/mL) and/or U60E (20 μg/mL) for 24 h. Nitric oxide (NO) production was determined by Griess assay. Values are represented as the mean of fold increase ± SD (*n* = 4). * *p* < 0.05.

We first investigated whether U60E affects the viability of ECs using an MTT assay. U60E had no effect on the of human umbilical vein endothelial cells (HUVECs) at concentrations up to 40 μg/mL (Figure 1A and Figure S1). *U. davidiana* was previously shown to reduce NO production in macrophages and microglia [29,30,31]; hence, we investigated how U60E affects the activity of eNOS, which induces NO production in ECs. U60E reduced the VEGF-mediated upregulation in the phosphorylation of eNOS (Figure 1B) and the basal level of eNOS phosphorylation in HUVECs in a dose-dependent manner (Figure 1B). At a concentration of 20 μg/mL, U60E most effectively reduced eNOS phosphorylation (Figure 1B). Hence, we chose this concentration for subsequent experiments. U60E not only reduced the basal level of eNOS phosphorylation in HUVECs but also decreased the VEGF-mediated upregulation in the phosphorylation of eNOS (Figure 1C–D). In addition, U60E reduced the basal level of NO production in HUVECs and suppressed the increased NO production by VEGF (Figure 1E). These results suggest that U60E inhibits the basal level of NO production and the increase in VEGF-induced NO production by suppressing the basal eNOS activity and the increased VEGF-induced eNOS activity in HUVECs, respectively.

### 2.2. U60E Inhibits VEGF-Induced Angiogenesis in HUVECs

**Figure 2:**
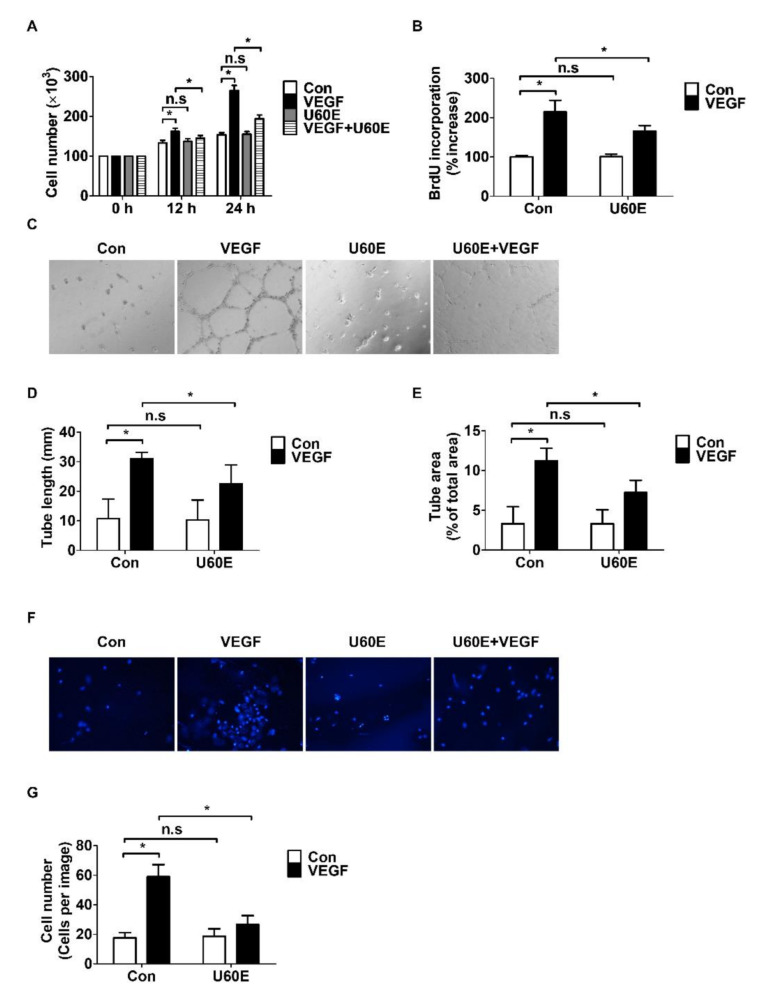
Effect of U60E on VEGF-induced angiogenesis of HUVECs. (**A**) Proliferation of HUVECs treated with VEGF (20 ng/mL) and/or U60E (20 μg/mL) for indicated times was determined by cell counting. The graph represents the means ± SD (*n* = 3). (**B**) HUVECs were treated with VEGF (20 ng/mL) and/or U60E (20 μg/mL) for 24 h. Cell proliferation was determined by a BrdU proliferation ELISA kit. Results are expressed as percentage increase in BrdU incorporation versus control value. Means ± SD (*n* = 4). (**C**) Representative images of tube formation by HUVECs treated with VEGF (20 ng/mL) and/or U60E (20 μg/mL) for 24 h. Original magnification ×40. (**D**,**E**) Quantitative analysis of tube lengths (mm) and tube area (% of total area) in (**C**) was performed. The bar graph represents the means ± SD (*n* = 4). (**F**) Representative images of cell migration of HUVECs treated with VEGF (20 ng/mL) and/or U60E (20 μg/mL) for 24 h. The migrated cells were stained with DAPI solution. Original magnification ×40. (**G**) Quantitative analysis of cell migration in (F) was performed. The bar graph represents the means ± SD (*n* = 4). n.s indicates *p* > 0.05, * *p* < 0.05.

To confirm whether U60E inhibited VEGF-induced angiogenesis in HUVECs, an in vitro angiogenesis assay evaluating cell proliferation, tube formation, and cell migration was performed. We determined the proliferation of HUVECs by cell counting and BrdU proliferation assays. U60E inhibited VEGF-induced HUVEC proliferation (Figure 2A,B) but was not involved in the proliferation of HUVECs in the absence of VEGF (Figure 2A,B). We analyzed the additional antiangiogenic effects of U60E by evaluating tube formation ability of HUVECs on Matrigel. U60E inhibited the tube length and area of HUVECs increased by VEGF (Figure 2C–E). However, U60E failed to affect the tube length and area of HUVECs untreated with VEGF (Figure 2C–E). We also analyzed the additional antiangiogenic effects of U60E by evaluating the migration ability of HUVECs through a transwell assay and wound-healing assay. U60E inhibited the migration of HUVECs increased by VEGF (Figure 2F,G and Figure S2A,B). However, U60E failed to affect the migration of HUVECs untreated with VEGF (Figure 2F,G and Figure S2A,B). These results indicate that U60E inhibits the VEGF-induced angiogenesis of HUVECs.

### 2.3. U60E Inhibits Angiogenesis by Suppressing VEGF-Mediated eNOS Activity

**Figure 3:**
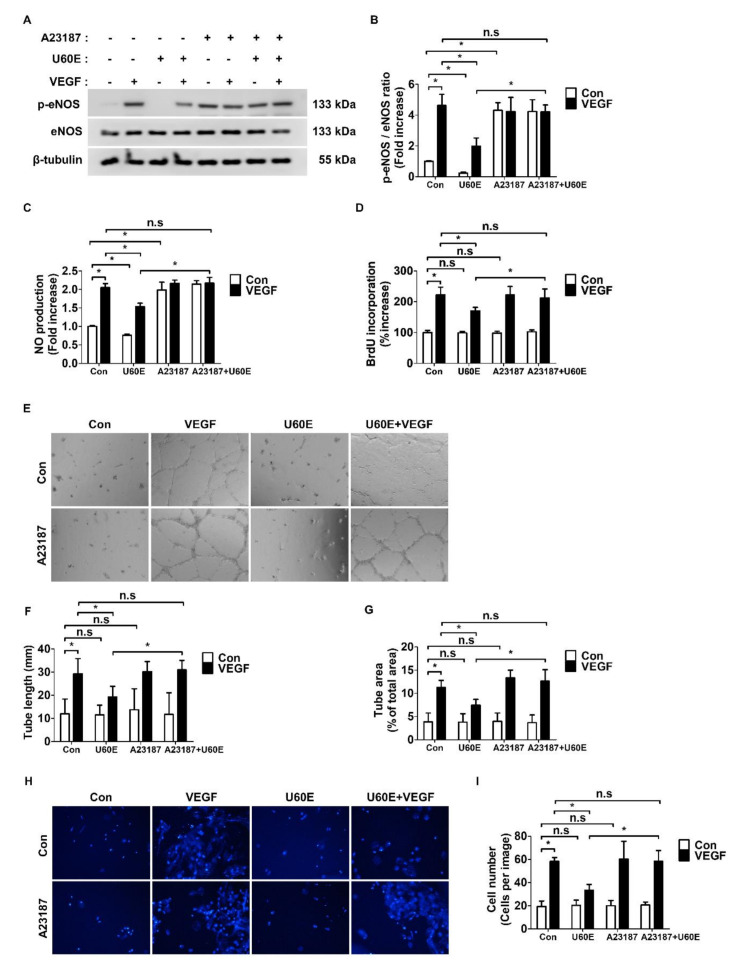
Effects of U60E-mediated decrease in eNOS activation and NO production on angiogenesis. (**A**) HUVECs were treated with VEGF (20 ng/mL), U60E (20 μg/mL), and/or A23187 (eNOS activator, 5 μM) for 30 min. The phosphorylation of eNOS (p-eNOS) was determined by Western blot analysis. eNOS and β-tubulin were used as controls. (**B**) Quantitative densitometric analysis of Western blots in (**A**). The bar graph represents the means ± SD (*n* = 3). (**C**) HUVECs were treated with VEGF (20 ng/mL), U60E (20 μg/mL), and/or A23187 (5 μM) for 24 h. The nitric oxide (NO) production was determined by Griess assay. Values are represented as the mean of fold increase ± SD (*n* = 4). (**D**) HUVECs were treated with VEGF (20 ng/mL), U60E (20 μg/mL), and/or A23187 (5 μM) for 24 h. Cell proliferation was determined by BrdU proliferation ELISA kit. Results are expressed as the percentage increase in BrdU incorporation versus control value. Means ± SD (*n* = 4). (**E**) Representative images of tube formation by HUVECs treated with VEGF (20 ng/mL), U60E (20 μg/mL), and/or A23187 (5 μM) for 24 h. Original magnification ×40. (**F**,**G**) Quantitative analysis of tube lengths (mm) and tube area (% of total area) in (**E**) was performed. The bar graph represents the means ± SD (*n* = 4). (**H**) Representative images of cell migration of HUVECs treated with VEGF (20 ng/mL), U60E (20 μg/mL), and/or A23187 (5 μM) for 24 h. The migrated cells were stained with DAPI solution. Original magnification ×40. (**I**) Quantitative analysis of cell migration in (**H**) was performed. The bar graph represents the means ± SD (*n* = 4). n.s indicates *p* > 0.05, * *p* < 0.05.

To determine if the inhibitory effect of U60E on VEGF-induced eNOS activity affects angiogenesis, we treated eNOS activator A23187 with HUVECs as well as another ECs—human retinal microvascular endothelial cells (HRMECs). Therefore, U60E no longer inhibited VEGF-induced eNOS phosphorylation and NO production (Figure 3A–C and Figure S3A–C). In addition, A23187 alone increased eNOS phosphorylation and NO production in both HUVECs and HRMECs (Figure 3A–C and Figure S3A–C). In both HUVECs and HRMECs treated with A23187, U60E failed to inhibit the VEGF-induced proliferation, tube formation, and migration (Figure 3D–I and Figure S3D–I). However, A23187 alone had no effect on the proliferation, tube formation, and migration ability of HUVECs and HRMECs (Figure 3D–I and Figure S3D–I). These observations suggest that U60E inhibits the VEGF-induced angiogenesis by suppressing eNOS activity and NO production.

### 2.4. U60E Inhibits eNOS Activity and NO Production by Reducing Akt Activity

**Figure 4:**
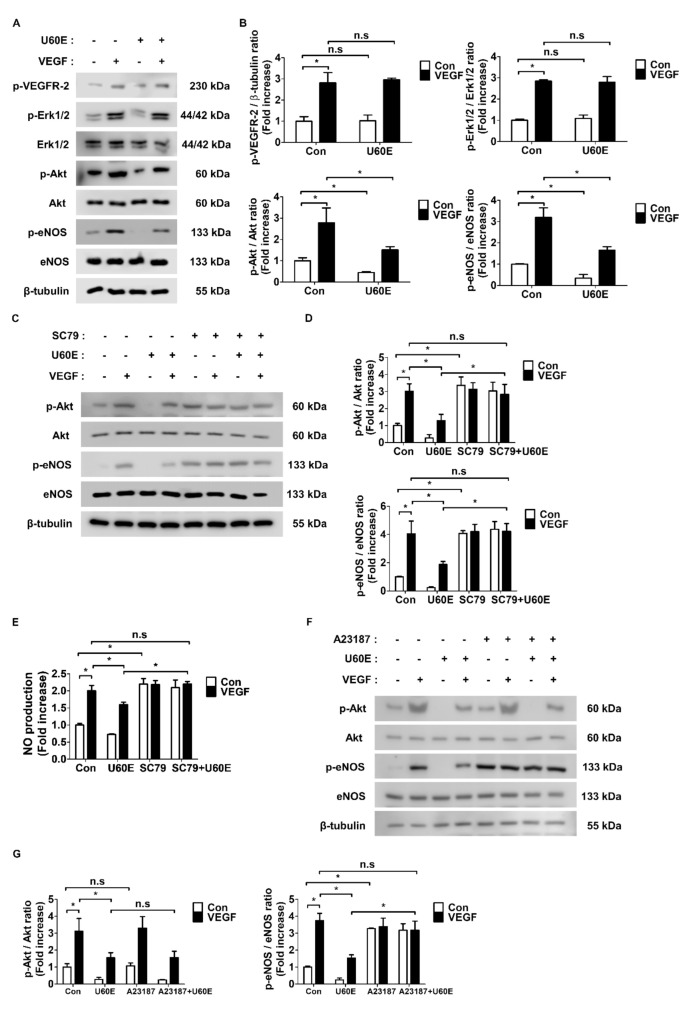
Involvement of Akt signaling in U60E-mediated decrease in eNOS activation and NO production in HUVECs. (**A**) HUVECs were treated with VEGF (20 ng/mL) and/or U60E (20 μg/mL) for 30 min. The phosphorylation of VEGFR-2 (p-VEGFR-2), Erk1/2 (p-Erk1/2), Akt (p-Akt), and eNOS (p-eNOS) was determined by Western blot analysis. Erk1/2, Akt, eNOS, and β-tubulin were used as controls. (**B**) Quantitative densitometric analysis of Western blots in (**A**). The bar graph represents the means ± SD (*n* = 3). (**C**) HUVECs were treated with VEGF (20 ng/mL), U60E (20 μg/mL), and/or SC79 (Akt activator, 1 μg/mL) for 30 min. The phosphorylation of Akt (p-Akt) and eNOS (p-eNOS) was determined by Western blot analysis. Akt, eNOS, and β-tubulin were used as controls. (**D**) Quantitative densitometric analysis of Western blots in (**C**). The bar graph represents the means ± SD (*n* = 3). (**E**) HUVECs were treated with VEGF (20 ng/mL), U60E (20 μg/mL), and/or SC79 (1 μg/mL) for 24 h. The nitric oxide (NO) production was determined by Griess assay. Values are represented as the mean of fold increase ± SD (*n* = 4). (**F**) HUVECs were treated with VEGF (20 ng/mL), U60E (20 μg/mL), and/or A23187 (eNOS activator, 5 μM) for 30 min. The phosphorylation of Akt (p-Akt) and eNOS (p-eNOS) was determined by Western blot analysis. Akt, eNOS, and β-tubulin were used as controls. (**G**) Quantitative densitometric analysis of Western blots in (**F**). The bar graph represents the means ± SD (*n* = 3). n.s indicates *p* > 0.05, * *p* < 0.05.

We next confirmed whether U60E affects other pathways induced by VEGF. As VEGF generally induces angiogenesis by activating VEGFR-2 and its downstream proteins Akt and Erk1/2, we investigated the effect of U60E on these protein activities. Interestingly, U60E had no effect on VEGFR-2 and Erk1/2 phosphorylation but reduced Akt phosphorylation (Figure 4A,B). We next used the Akt activator SC79 and the eNOS activator A23187 to determine any association between U60E-mediated decrease in Akt activity and eNOS activity. Interestingly, U60E no longer inhibited VEGF-induced eNOS phosphorylation and NO production in both HUVECs and HRMECs treated with SC79 (Figure 4C–E and Figure S4A–C). Further, SC79 treatment alone increased eNOS phosphorylation and NO production in both HUVECs and HRMECs (Figure 4C–E and Figure S4A–C). However, treatment of HUVECs and HRMECs with A23187 had no effect on VEGF-induced Akt phosphorylation inhibited by U60E (Figure 4F,G and Figure S4D,E). A23187 alone did not affect Akt phosphorylation in both HUVECs and HRMECs (Figure 4F,G and Figure S4D,E). Thus, U60E may inhibit VEGF-induced Akt activity in ECs, thereby suppressing eNOS activity and NO production and preventing angiogenesis.

## 3. Discussion

Abnormal angiogenesis is associated with exacerbation of various diseases such as cancer, DR, and ROP, necessitating precise control over this process. The VEGF signaling pathway is closely related to the induction of angiogenesis in vascular ECs [6,7]. VEGF binds to VEGFR-2 among VEGF receptors with very high affinity and activates the downstream signaling. Therefore, VEGF is involved in mediating major changes in ECs, such as angiogenesis [32,33,34], and targeting the VEGF-associated signaling pathway may alleviate various symptoms by effectively regulating angiogenesis [35,36]. Many drugs that inhibit angiogenesis by targeting VEGF-related pathways have been discovered. Bevacizumab, a VEGF protein-neutralizing antibody, is known to effectively inhibit angiogenesis in various cancers and DR [37,38,39]. In addition, tyrosine kinase inhibitors such as sunitinib and sorafenib inhibit angiogenesis by suppressing VEGF receptor tyrosine kinases and are also known to be effective against various cancers and ocular diseases [40,41,42,43,44,45]. However, these drugs are expensive or cause serious toxicities [46]. Therefore, it is imperative to develop VEGF pathway inhibitors that are affordable and without/minimal side effects. Interestingly, natural products are attracting attention as major compounds for the development of new drugs because they not only have low prices and low side effects, but also have high chemical diversity and biochemical specificity [47]. In fact, drugs such as penicillin, aspirin, and veregen, which are widely used in clinical practice, are representative pharmaceuticals developed from extracts of natural products [48]. Therefore, we focused on research on the inhibition of angiogenesis induced by VEGF using the extracts of natural products.

*U. davidiana* is a natural product traditionally consumed for its pharmacological properties such as antioxidant, anti-inflammatory, and anticancer effects [24,25,26]. Interestingly, *U. davidiana* was previously known to inhibit chorioallantoic membrane angiogenesis [28]. Therefore, we performed in vitro experiments to confirm whether U60E directly inhibits angiogenesis in ECs. However, U60E had no direct effects on cell viability except at concentrations above 80 μg/mL, which induced apoptosis of HUVECs (Figure 1A and Figure S1A,B). In addition, U60E alone did not affect the proliferation, tube formation, or migration of HUVECs and HRMECs (Figure 2 and Figures S2 and S3). *U. davidiana* was previously shown to reduce NO production in macrophages and microglia [29,30]. In addition, VEGF treatment induced eNOS activation in ECs, resulting in increased NO production and angiogenesis [49]. Therefore, we hypothesized that U60E may play an important role by inhibiting VEGF-induced angiogenesis through the reduction in VEGF-mediated eNOS activation or NO production. We confirmed that U60E inhibited angiogenesis by reducing eNOS activation and NO production induced by VEGF (Figure 3A–I and Figure S3A–I). On the other hand, U60E decreased eNOS activation and NO production in both HUVECs and HRMECs in the absence of VEGF but had no influence on proliferation, tube formation, or migration (Figure 3A–I and Figure S3A–I). The eNOS activator A23187 in the absence of VEGF increased the production of eNOS and NO in both HUVECs and HRMECs without affecting their proliferation, tube formation, and migration (Figure 3A–I and Figure S3A–I). Therefore, these results suggest that NO production in ECs under angiogenic conditions, such as in the presence of VEGF, plays an important role in promoting angiogenesis and that U60E inhibits angiogenesis by reducing VEGF-induced NO production in ECs.

In the present study, we demonstrated the role of U60E in reducing angiogenesis by blocking VEGF-induced Akt activation. It was previously known that VEGF activates VEGFR-2 and induces angiogenesis through the Akt and Erk pathways [9,10,11]. Here, we found that U60E did not affect VEGFR-2 and Erk activation induced by VEGF but decreased Akt activation (Figure 4A,B). As U60E reduced both Akt and eNOS activation in HUVECs (Figure 4A,B) and the Akt-eNOS-NO pathway was known to be involved in this phenomenon [50,51,52], we hypothesized that U60E reduces the VEGF-mediated Akt activation and suppressed eNOS activation and NO production, thereby inhibiting angiogenesis. Treatment with SC79, an Akt activator, abrogated the effect of U60E on VEGF-induced eNOS activation and NO production in both HUVECs and HRMECs (Figure 4C–E and Figure S4A–C). On the other hand, treatment with A23187, an eNOS activator, had no influence on the effect of U60E on VEGF-induced Akt activation in both HUVECs and HRMECs (Figure 4F,G and Figure S4D,E). Therefore, these results suggest that U60E reduces eNOS activation and NO production by suppressing VEGF-activated Akt, thereby preventing angiogenesis.

In summary, our study demonstrates the effect of U60E on EC proliferation, tube formation, and migration ability. We show that U60E inhibited angiogenesis induced by VEGF by blocking the Akt/eNOS/NO pathway. Together, these results suggest the potential therapeutic benefit of U60E for inhibition of VEGF-induced angiogenesis in proangiogenic diseases. Although this study did not identify the major chemical component of U60E, which inhibits VEGF-induced angiogenesis, research related to this is expected to be an important study that can more clearly develop new angiogenesis inhibitors in the future.

## 4. Materials and Methods

### 4.1. Cell Cultures

Human umbilical vein endothelial cells (HUVECs, Lonza, Rockland, ME, USA), and human retina microvascular endothelial cells (HRMECs, ACBRI, Kirkland, WA, USA) were maintained in M199 (HyClone, Logan, UT, USA) medium supplemented with 20% fetal bovine serum (FBS). Cells were cultured at 37°C in an incubator with a humidified atmosphere of 5% CO_2_.

### 4.2. Reagents and Antibodies

Recombinant human VEGF was purchased from R&D Systems (Minneapolis, MA, USA), and the eNOS activator A23187 was obtained from Millipore-Sigma (St. Louis, MO, USA). The Akt activator SC79 was supplied by Selleck Chemicals (Houston, TX, USA). Primary anti-phospho-eNOS, anti-eNOS, anti-phospho-VEGFR-2, anti-phospho-Erk1/2, anti-Erk1/2, anti-phospho-Akt, and anti-Akt antibodies were procured from Cell Signaling Technology. Anti-β-tubulin and peroxidase-conjugated secondary antibodies were provided by Santa Cruz Biotechnology (Dallas, TX, USA).

### 4.3. Preparation of U60E Extracts

*U. davidiana* was collected at 10 kg (branches with barks) from Dolsan-eup, Yeosu-si, Jeollanam-do, Republic of Korea, in June 2020. Certification was obtained and 10 kg of a voucher specimen (UDB2020-06) was extracted once with a 60% edible ethanol extract at room temperature. Ethanol was evaporated by vacuum concentration to obtain an extract with a yield of 570 g. The dried 60% edible ethanol extract was dissolved in dimethyl sulfoxide (DMSO) and diluted in cell culture medium. The final concentration of DMSO in cell culture medium was less than 0.01%.

### 4.4. Cell Viability Assay

Cell viability was evaluated with the 3-(4,5-di methylthiazol-2-yl)-2,5-diphenyltetrazolium bromide (MTT) labeling kit (Millipore-Sigma). In brief, 5 × 10^3^ cells were seeded into 96-well plates for 24 h and treated with U60E. The cells were then incubated with the MTT reagent (5 mg/mL) for 3 h, and the formazan product formed was evaluated by measuring absorption intensity at 570 nm (Tecan, Männedorf, Switzerland) wavelength.

### 4.5. Western Blot Analysis

The cells were lysed in a lysis buffer containing 20 mM Tris (pH 7.5), 150 mM sodium chloride (NaCl), 1% Triton X-100, and a protease inhibitor cocktail. Cell lysates were separated on 7–10% sodium dodecyl sulfate polyacrylamide gel electrophoresis gels and the separated proteins were transferred onto nitrocellulose membranes. The blots were incubated with indicated primary antibodies (1:1000) at 4 °C for overnight and then probed with secondary antibodies (1:5000) at room temperature for 1 h. The blots were incubated with an enhanced chemiluminescence substrate (Thermo Fisher Scientific, Waltham, MA, USA) and exposed to film.

### 4.6. BrdU Enzyme-Linked Immunosorbent Assay (ELISA) for Cell Proliferation Estimation

To measure cell proliferation, the Cell Proliferation BrdU ELISA kit (Roche Diagnostics, Indianapolis, IN, USA) was used according to the manufacturer’s protocol. Cells treated with the indicated reagents for 24 h were labeled with 10 μM BrdU for 1 h, and then incubated with the anti-BrdU peroxidase-conjugated antibody for 90 min. After washing, the bound peroxidase was detected by the substrate reaction, which was measured on an ELISA plate reader (Tecan) at 450 nm wavelength.

### 4.7. Cell Counting

To measure the number of cells, 10^5^ cells were seeded in the presence of indicated reagents. At 0, 12, and 24 h after treatment, viable cells were counted using a hemocytometer following trypan blue staining (Millipore-Sigma).

### 4.8. Tube Formation

To evaluate tube formation, 200 μL of growth factor-reduced Matrigel (Corning Inc., NY, USA) was coated onto 24-well plates at 37 °C for 30 min. Cells (5 × 10^4^) were then seeded into the coated plates in the presence of indicated reagents and incubated for 24 h. Tube length and area were quantified using the ImageJ software (NIH, Bethesda, MD, USA).

### 4.9. Transwell Cell Migration Assay

Cells were plated on the upper chambers of transwells with 8 μm pores and then inserted into 24 well plates. Next, 6 × 10^3^ cells were seeded into the upper chambers (Corning Inc.) for 6 h and starved with media containing 1% FBS for 12 h. Cells were then treated with the indicated agents and allowed to migrate through the pores for another 24 h. The membrane in the upper chamber was then separated, washed with PBS and stained with DAPI solution (Millipore-Sigma). For quantitative analysis, the number of cells at four sites randomly selected on one membrane was counted and the values of four different membranes were quantified.

### 4.10. NO Production Measurement

NO concentrations in cells were determined by detecting the concentration of nitrite, the stable product of NO. Cells (5 × 10^4^) were seeded in 24-well plates with the indicated reagents for 24 h. The medium was collected and NO content in the medium was measured based on the Griess reaction using a NO assay kit (Millipore-Sigma). The optical densities at a wavelength of 540 nm were obtained using a microplate reader (Tecan), and NO concentration was calculated according to the calibration curve.

### 4.11. Statistics Analysis

Statistical analyses were performed using a standard two-tailed Student’s *t*-test assuming unequal variances, and *p* < 0.05 was considered statistically significant. Quantitative data and figures are presented as mean ± standard deviation (SD).

## Data Availability

Data sharing not applicable.

## Supplementary Materials

Figure S1. Effect of *U. davidiana* (U60E) on the survival of endothelial cell (ECs). Human Umbilical Vein Endothelial Cells (HUVECs) were treated with U60E for 24 h at indicated doses. (A) The cell viability was determined by MTT assay. The bar graph represents the means ± SD (*n* = 4). (B) Cell apoptosis was analyzed using the Annexin V/propidium iodide stain-ing and flow cytometric analysis. The apoptotic cells were expressed as percentage of apoptotic cells versus total cell population. Means ± SD (*n* = 3). * *p* < 0.05, Figure S2. Effect of U60E on Vas-cular endothelial growth factor (VEGF)-induced migration in HUVECs. (A) Representative images of wound healing assay of HUVECs treated with VEGF (20 ng/ml) and/or U60E (20 μg/ml) at 0 and 24 h. Original magnification ×40. (B) The number of cells migrating into the initial wound ar-ea was counted at 24 h. The bar graph represents the means ± SD (*n* = 4). n.s indicates *p* > 0.05, * *p* < 0.05, Figure S3. Effects of U60E-mediated decrease in endothelial nitric oxide synthase (eNOS) activation and nitric oxide (NO) production on angiogenesis. (A) Human retinal microvascular endothelial cells (HRMECs) were treated with VEGF (20 ng/mL), U60E (20 μg/mL), and/or A23187 (eNOS activator, 5 μM) for 30 min. The phosphorylation of eNOS (p-eNOS) was deter-mined by Western blot analysis. eNOS and β-tubulin were used as controls. (B) Quantitative den-sitometric analysis of Western blots in (A). The bar graph represents the means ± SD (*n* = 3). (C) HRMECs were treated with VEGF (20 ng/mL), U60E (20 μg/mL), and/or A23187 (5 μM) for 24 h. The nitric oxide (NO) production was determined by Griess assay. Values are represented as the mean of fold increase ± SD (*n* = 4). (D) HRMECs were treated with VEGF (20 ng/mL), U60E (20 μg/mL), and/or A23187 (5 μM) for 24 h. Cell proliferation was determined by BrdU proliferation ELISA kit. Results are expressed as the percentage increase in BrdU incorporation versus control value. Means ± SD (*n* = 4). (E) Representative images of tube formation by HRMECs treated with VEGF (20 ng/mL), U60E (20 μg/mL), and/or A23187 (5 μM) for 24 h. Original magnification ×40. (F-G) Quantitative analysis of tube lengths (mm) and tube area (% of total area) in (E) was per-formed. The bar graph represents the means ± SD (*n* = 4). n.s indicates *p* > 0.05, * *p* < 0.05. (H) Representative images of cell migration of HRMECs treated with VEGF (20 ng/mL), U60E (20 μg/mL), and/or A23187 (5 μM) for 24 h. The migrated cells were stained with DAPI solution. Original magnification ×40. (I) Quantitative analysis of cell migration in (H) was performed. The bar graph represents the means ± SD (*n* = 4). n.s indicates *p* > 0.05, * *p* < 0.05, Figure S4. Involve-ment of Akt signaling in U60E-mediated decrease in eNOS activation and NO production in HRMECs. (A) HRMECs were treated with VEGF (20 ng/mL), U60E (20 μg/mL), and/or SC79 (Akt activator, 1 μg/mL) for 30 min. The phosphorylation of Akt (p-Akt) and eNOS (p-eNOS) was de-termined by Western blot analysis. Akt, eNOS, and β-tubulin were used as controls. (B) Quantita-tive densitometric analysis of Western blots in (A). The bar graph represents the means ± SD (*n* = 3). (C) HRMECs were treated with VEGF (20 ng/mL), U60E (20 μg/mL), and/or SC79 (1 μg/mL) for 24 h. The nitric oxide (NO) production was determined by Griess assay. Values are represent-ed as the mean of fold increase ± SD (*n* = 4). (D) HRMECs were treated with VEGF (20 ng/mL), U60E (20 μg/mL), and/or A23187 (eNOS activator, 5 μM) for 30 min. The phosphorylation of Akt (p-Akt) and eNOS (p-eNOS) was determined by Western blot analysis. Akt, eNOS, and β-tubulin were used as controls. (E) Quantitative densitometric analysis of Western blots in (D). The bar graph represents the means ± SD (*n* = 3). n.s indicates *p* > 0.05, * *p* < 0.05.

## Sample Availability

Samples of the compounds: a 60% edible ethanolic extract of *U. davidiana* are available from the authors.
