# Supplementary material for: A 60% Edible Ethanolic Extract of Ulmus davidiana Inhibits Vascular Endothelial Growth Factor-Induced Angiogenesis
Source: Molecules. 2021 Feb 3;26(4):781. doi: 10.3390/molecules26040781 (PMC7913375; doi:10.3390/molecules26040781)
Supplement: Supplementary file 1 [file molecules-26-00781-s001.pdf]

Supporting Materials

A 60% Edible Ethanolic Extract of *Ulmus Davidiana* Inhibits Vascular Endothelial Growth Factor-Induced Angiogenesis

Jeongho Park [1,#], Hyun-Ouk Kim [2,3,#], Kwang-Hyun Park [4,5,#], Myung-Bok Wie[1], Sun-Eun Choi [6,*] and Jang-Hyuk Yun [1,*]

[1] College of Veterinary Medicine and Institute of Veterinary Science, Kangwon National University, Chuncheon, Gangwon, Republic of Korea ; jhp@kangwon.ac.kr (J.P.); mbwie@kangwon.ac.kr (M.-B.W)
[2] Department of Biotechnology and Bioengineering, Kangwon National University, Chuncheon, Gangwon-do 24341, Republic of Korea ; kimhoman@kangwon.ac.kr
[3] Biohealth-machinery Convergence Engineering, Kangwon National University, Chuncheon, Gangwon-do 24341, Republic of Korea
[4] Department of Emergency Medical Rescue, Nambu University, Gwangju 62271, Republic of Korea ; khpark@nambu.ac.kr
[5] Department of Emergency Medicine, Graduate School of Chonnam National University Gwangju 61469, Republic of Korea
[6] Department of Forest Biomaterials Engineering, Kangwon National University 24341, Republic of Korea.
[#] These authors contributed equally to this work.
* Correspondence: Correspondence: oregonin@kangwon.ac.kr (S.-E.C.); yunjh@kangwon.ac.kr (J.-H.Y.); Tel.: 82-33-250-8324 (S.-E.C.); 82-33-250-8653 (J.-H.Y.)

1. Material and Methods

2.1. FACS Analysis

In order to evaluate apoptosis, 5×10^5 cells were treated with U60E at 37°C for 24 h. The cells were harvested and washed twice in PBS. The cells were stained with FITC annexin V and PI for 15 min, and analyzed by flow cytometry on the FACS Calibur (BD Biosciences) and data were analyzed with Flowjo software. Annexin V positive cells were determined to be apoptotic cells.

2.2. Wound-Healing Migration Assay

Cells were seeded in 24-well plates and grown to 80% confluence. Wounds were created by scraping monolayer cells with a 200 µl pipette tip, and nonadherent cells were washed off with medium. The cells were allowed to migrate for 24 h in the presence of indicated reagents, and then their migration was examined. In the images, the edge of the initial wound area is marked by lines. The edge of the initial wound area was overlaid with the image taken at 24 h after scratching. The number of cells migrating into the initial wound area was counted at 24 h after scratching.

2. Figure legends

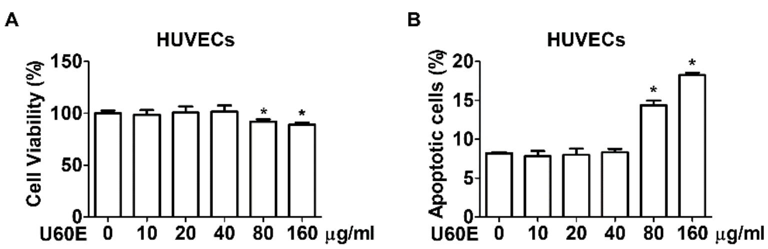

Figure S1. Effect of *U. davidiana* (U60E) on the survival of endothelial cell (ECs). Human Umbilical Vein Endothelial Cells (HUVECs) were treated with U60E for 24 h at indicated doses. (A) The cell viability was determined by MTT assay. The bar graph represents the means ± SD (n = 4). (B) Cell

apoptosis was analyzed using the Annexin V/propidium iodide staining and flow cytometric analysis. The apoptotic cells were expressed as percentage of apoptotic cells versus total cell population. Means ± SD (n = 3). *P < 0.05.

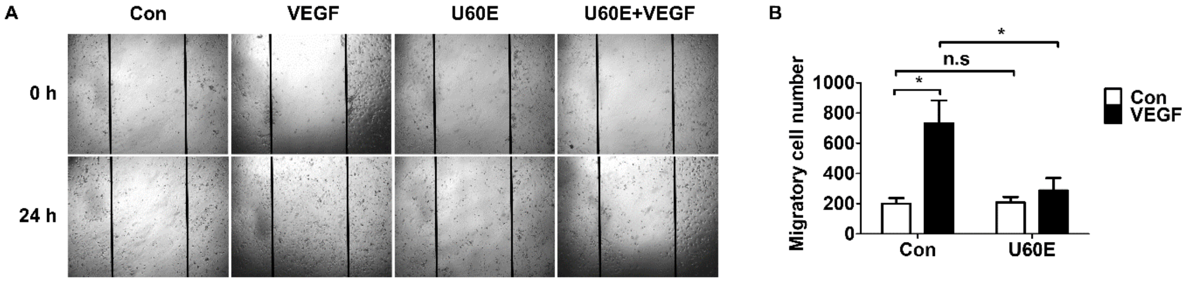

Figure S2. Effect of U60E on Vascular endothelial growth factor (VEGF)-induced migration in HUVECs. (A) Representative images of wound healing assay of HUVECs treated with VEGF (20 ng/ml) and/or U60E (20 μg/ml) at 0 and 24 h. Original magnification ×40. (B) The number of cells migrating into the initial wound area was counted at 24 h. The bar graph represents the means ± SD (n = 4). n.s indicates P > 0.05, *P < 0.05.

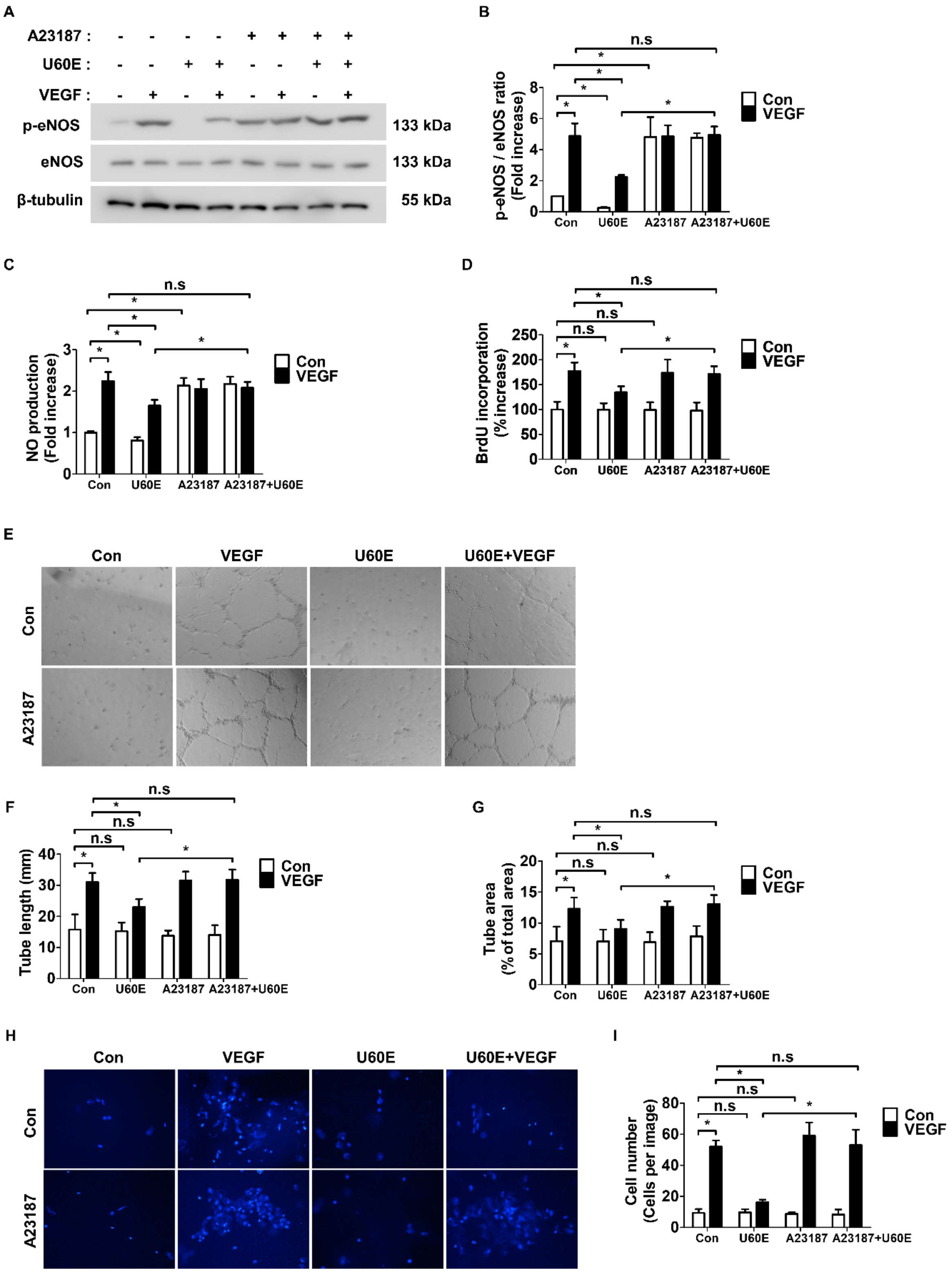
A
A23187 :
U60E :
VEGF :
p-eNOS
eNOS
β-tubulin
133 kDa
133 kDa
55 kDa
B
p-eNOS / eNOS ratio (Fold increase)
Con
VEGF
Con U60E A23187 A23187+U60E
n.s
C
NO production (Fold increase)
Con
VEGF
Con U60E A23187 A23187+U60E
n.s
D
BrdU incorporation (% increase)
Con
VEGF
Con U60E A23187 A23187+U60E
n.s
E
Con VEGF U60E U60E+VEGF
Con
A23187
F
Tube length (mm)
Con
VEGF
Con U60E A23187 A23187+U60E
n.s
G
Tube area (% of total area)
Con
VEGF
Con U60E A23187 A23187+U60E
n.s
H
Con VEGF U60E U60E+VEGF
Con
A23187
I
Cell number (Cells per image)
Con
VEGF
Con U60E A23187 A23187+U60E
n.s

Figure S3. Effects of U60E-mediated decrease in endothelial nitric oxide synthase (eNOS) activation and nitric oxide (NO) production on angiogenesis. (A) Human retinal microvascular endothelial cells (HRMECs) were treated with VEGF (20 ng/mL), U60E (20 μg/mL), and/or A23187 (eNOS activator, 5 μM) for 30 min. The phosphorylation of eNOS (p-eNOS) was determined by Western blot analysis. eNOS and β-tubulin were used as controls. (B) Quantitative densitometric analysis of Western blots in (A). The bar graph represents the means ± SD (n = 3). (C) HRMECs were treated with VEGF (20 ng/mL), U60E (20 μg/mL), and/or A23187 (5 μM) for 24 h. The nitric oxide (NO) production was determined by Griess assay. Values are represented as the mean of fold increase ± SD (n = 4). (D) HRMECs were treated with VEGF (20 ng/mL), U60E (20 μg/mL), and/or A23187 (5 μM) for 24 h. Cell proliferation was determined by BrdU proliferation ELISA kit. Results are expressed as the percentage increase in BrdU incorporation versus control value. Means ± SD (n = 4). (E) Representative images of tube formation by HRMECs treated with VEGF (20 ng/mL), U60E (20 μg/mL), and/or A23187 (5 μM) for 24 h. Original magnification ×40. (F-G) Quantitative analysis of tube lengths (mm) and tube area (% of total area) in (E) was performed. The bar graph represents the means ± SD (n = 4). n.s indicates $P > 0.05$, *$P < 0.05$. (H) Representative images of cell migration of HRMECs treated with VEGF (20 ng/mL), U60E (20 μg/mL), and/or A23187 (5 μM) for 24 h. The migrated cells were stained with DAPI solution. Original magnification ×40. (I) Quantitative analysis of cell migration in (H) was performed. The bar graph represents the means ± SD (n = 4). n.s indicates $P > 0.05$, *$P < 0.05$.

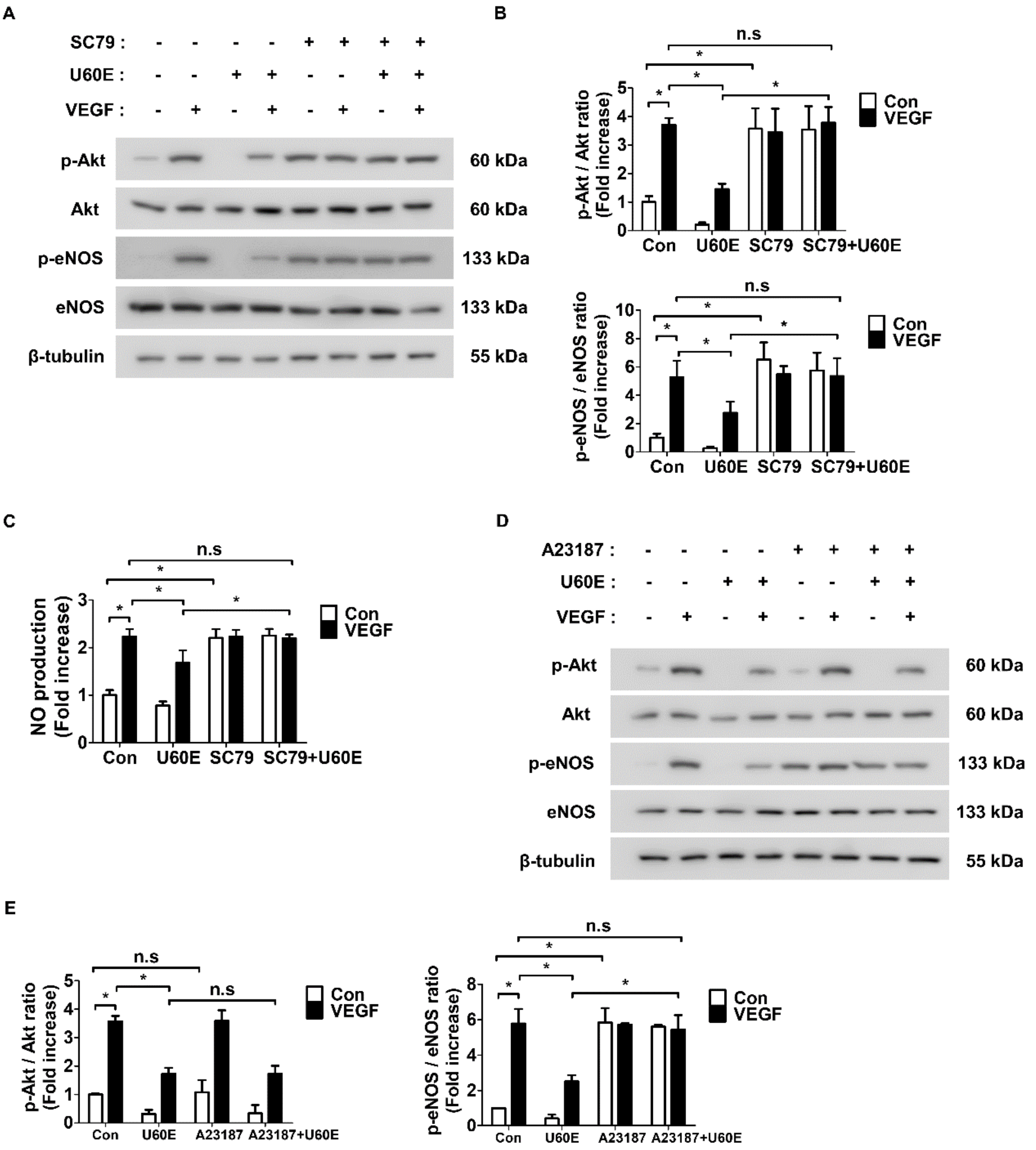
A
SC79 : - - - - + + + +
U60E : - - + + - - + +
VEGF : - + - + - + - +
p-Akt
60 kDa
Akt
60 kDa
p-eNOS
133 kDa
eNOS
133 kDa
β-tubulin
55 kDa
B
p-Akt / Akt ratio (Fold increase)
Con
VEGF
Con U60E SC79 SC79+U60E
n.s
*
p-eNOS / eNOS ratio (Fold increase)
C
NO production (Fold increase)
D
A23187 : - - - - + + + +
U60E : - - + + - - + +
VEGF : - + - + - + - +
E
Con U60E A23187 A23187+U60E

Figure S4. Involvement of Akt signaling in U60E-mediated decrease in eNOS activation and NO production in HRMECs. (A) HRMECs were treated with VEGF (20 ng/mL), U60E (20 μg/mL), and/or SC79 (Akt activator, 1 μg/mL) for 30 min. The phosphorylation of Akt (p-Akt) and eNOS (p-eNOS) was determined by Western blot analysis. Akt, eNOS, and β-tubulin were used as controls. (B) Quantitative densitometric analysis of Western blots in (A). The bar graph represents the means ± SD (n = 3). (C) HRMECs were treated with VEGF (20 ng/mL), U60E (20 μg/mL), and/or SC79 (1 μg/mL) for 24 h. The nitric oxide (NO) production was determined by Griess assay. Values are represented as the mean of fold increase ± SD (n = 4). (D) HRMECs were treated with VEGF (20 ng/mL), U60E (20 μg/mL), and/or A23187 (eNOS activator, 5 μM) for 30 min. The phosphorylation of Akt (p-Akt) and eNOS (p-eNOS) was determined by Western blot analysis. Akt, eNOS, and β-tubulin were used as controls. (E) Quantitative densitometric analysis of Western blots in (D). The bar graph represents the means ± SD (n = 3). n.s indicates $P > 0.05$, *$P < 0.05$.
